# The American International Congress on Tuberculosis

**Published:** 1907-10

**Authors:** 


### Communications.

### The American International Congress on Tuberculosis.

A meeting of the Council of the Congress was held on August 27, 1907, at the New York Press Club, 120 Nassau street, with a very full representation.

Dr. Matthew M. Smith, the Secretary of the Congress, late of Austin, but now of Dallas, Texas, was present and represented Dr. Irion, President of the Congress and President of the State Board of Health of the State of Louisiana, who, he said, strongly favored a rousing meeting of the Congress June 1, 1908, in the city of Chicago.

Dr. Smith strongly favored the same action and spoke in favor of it.

Dr. Nicholas Senn, of Chicago, was elected to fill the vacancy in the Council, caused by the death of Dr. A. P. Grinnell. Mr. Clark Bell, who acted as Secretary of the Council and as proxy for Dr. A. C. Smith, President of the State Board of Health. of Oregon; Dr. N. K. Foster, Secretary of the State Board of Health of California, explained the deep interest felt in the work by both these gentlemen, who were unable to attend the session of the Council, but pledged their ardent and enthusiastic support to the Congress of June 1, 1908, at Chicago.

Mr. Bell also assured the Council of the warm aid and support of Dr. Morrow, late Secretary of the State Board of Health of Missouri; of Colonel S. L. Bean, of El Paso, Texas, for whom he also held proxies, and of the officers and Vice-Presidents, who were unable to be present at the Council meeting, two others of whom, Dr. Morrow, of Missouri, Dr. Thomas Bassett Keyes, of Chicago, and Dr. S. L. Bean, of El Paso, Texas, of their warm personal interest in the success of the Congress.

He made a memorial address in the life and career of Dr. A. P. Grinnell, as did Judge Wm. H. Francis, of New Jersey, a member of the Council.

The Council unanimously directed the executive officers to call a meeting of the body for June 1, 1908, at Chicago, and placed the local arrangements for the session in charge of a select committee composed of Surgeon General Nicholas Senn, M. D., Denslow Lewis, M. D., and Dr. Thomas Bassett Keyes, all of Chicago, with very full powers.

Dr. Juan J. De Ulloa, Consul General for Costa Rica, a member of the Council, who was present, was elected Secretary of the Congress for the Latin-speaking countries.

He was instructed to make an appeal to all the countries of the Western Hemisphere along the lines of the work of the body, followed in the Congress of 1906, held at the city of New York, soliicting the cordial support of men of all professions in those countries and bodies to aid and co-operate in the movement.

The Council unanimously instructed the President, Dr. Irion, of Louisiana, to make an appeal to the State and municipal boards of health to co-operate in the work of the Congress, and to contribute papers to the session. On the ways and means, the Council organized a committee to provide funds by appeals to all philanthropic and sympathetic persons to contribute financially to the expenses of the Congress, aiding in publishing the *Bulletin* of 1906, and meeting its past and future obligations.

The chairman named as that committee Dr. Juan J. De Ulloa, Chairman; Clark Bell, Esq., ex-Chairman Executive Committee, and Judge William H. Francis, of New Jersey.

Action was taken to organize a strong executive committee and the other committees to make, if possible, a still more successful meeting than that of the Congress of 1906, and to interest all those who desire the success of the Congress; also to interest the Governors of States not now identified with its work, State and other societies and organizations, legal, medical, scientific or fraternal to unite in the work in which men and women of all professions should be invited and encouraged to unite and co-operate.

A list of the officers of the Congress will be sent out with its appeal and the literature of the Congress, and members were asked to apply for the Bulletin of the New York Congress of 1906, now nearly completed and containing more than 200 pages of printed matter to the *Medico-Legal Journal,* or any of the officers of the society.

Mr. Bell authorized the Council in aid of the work to announce that Volume 25 of the *Medico-Legal Journal* would be sent half-price to all officers and delegates to the present Congress or the next, if paid for in advance.

The Council adjourned subject to the call of the chairman of the council.

---

HAHNEMANN MEDICAL COLLEGE OF THE PACIFIC
SACRAMENTO AND MAPLE STREETS
*Office of Dean*
DR. JAMES W. WARD
1380 SUTTER STREET

SAN FRANCISCO, CAL., September 17, 1907.

*Texas Medical Journal, Austin, Texas.*

GENTLEMEN: Your most valuable journal comes to the College Library regularly. It seems to me that your thoughtfulness is worthy of our most cordial thanks, and I further say that it has been of great value to the student body. I sincerely hope that you may profit by the influence cast before the students, and I am sure they have been reading its pages thoroughly.

Very sincerely yours,
DR. JAMES W. WARD.

---

NEW YORK, September 12, 1907.

*Editor Texas Medical Journal.*

DEAR SIR: We received a request from the Ways and Means Committee of the National Association of Retail Druggists to subscribe to the fund for the entertainment of the members of the N. A. R. D. at its forthcoming convention in the city of Chicago. In view of previous experience, it occurred to us to take steps to ascertain to what extent that "community of interest" expressly implied and naturally anticipated did actually prevail. We, therefore, caused prescriptions in which essence of pepsin, Fairchild's, was plainly specified, to be sent to the following mentioned drug stores: I. M. Light, 143 E. 35th St.; Herman Lieberman, 528 So. Halsted St.; J. S. Link, 649 W. 21st St.; C. P. Girten, 7100 Harvard Ave.; H. J. Holthoefer, N. W. cor. State and 32d Sts.; John J. Boehm, 748 So. Halsted St.; William W. Klore, 2354 State St.; William P. Knoche, N. E. cor. 61st and Halsted Sts.; Moyen Bros. (Geo. and F. W. Moyen), 1595 Milwaukee Ave, cor. Armitage.

Each vial so dispensed was upon receipt immediately sealed in its original wrapper and delivered to us, with complete data in every detail from the writing to the dispensing and sealing of the prescription. Upon examination, the fluid dispensed by each of these druggists was found to be a substitute—some preparation not made by us and not Fairchild's essence.

These substitutes were unlike in composition; they were of varying degrees of inferiority *and some practically worthless* for the purpose for which Fairchild's essence of pepsin is esteemed and employed.

There is one significant fact that should also be mentioned, and that is that the price at which the substitutes were sold is that charged the patient by pharmacists who dispense Fairchild's essence of pepsin when it is ordered.

We, therefore, bring these facts to your attention, again stating our intention to fight against substitution and for a "square deal."

Very truly yours,

Fairchild Bros. & Foster.

[And the *Red Back* is enlisted for "endurin' the war." Cut out the dispensing druggist and dispense your own drugs. Carry a case like the homo's do.]

